# Estimating within-stride metabolic cost from stride-average data using autoencoders and expander networks

**DOI:** 10.3389/fbioe.2025.1579085

**Published:** 2025-06-20

**Authors:** Manal Mustafa, Alex C. Dzewaltowski, Philippe Malcolm, Keegan J. Moore

**Affiliations:** ^1^ Department of Mechanical and Materials Engineering, University of Nebraska-Lincoln, Lincoln, NE, United States; ^2^ Department of Biomechanics, University of Nebraska Omaha, Omaha, NE, United States; ^3^ Scholl College of Podiatric Medicine, Rosalind Franklin University of Medicine and Science, North Chicago, IL, United States; ^4^ Daniel Guggenheim School of Aerospace Engineering, Georgia Institute of Technology, Atlanta, GA, United States

**Keywords:** walking, biomechanics, energetics, machine learning, system identification

## Abstract

**Introduction:**

Biomechanical changes due to aging increase the oxygen consumption of walking by over 30%. When this is coupled with reduced oxygen uptake capacity, the ability to sustain walking becomes compromised. This reduced physical activity and mobility can lead to further physical degeneration and mortality. Unfortunately, the underlying reasons for the increased metabolic cost are still inadequately understood. While motion capture systems can measure signals with high temporal resolution, it is impossible to directly characterize the fluctuation of metabolic cost throughout the gait cycle.

**Methods:**

To address this issue, this research focuses on computing the metabolic cost time series from the mean value using two neural-network-based approaches: autoencoders (AEs) and expanders. For the AEs, the encoders are designed to compress the input time series down to their mean value, and the decoder expands those values into the time series. After training, the decoder is extracted and applied to mean metabolic cost values to compute the time series. A second approach leverages an expander to map the mean values to the time series without an encoder. The networks are trained using ten different metabolic cost models generated by a computational walking model that simulates the gait cycle subjected to 35 different robotic perturbations without using experimental input data. The networks are validated using the estimated metabolic costs for the unperturbed gait cycle.

**Results:**

The investigation found that AEs without tied weights and the expanders performed best using nonlinear activation functions, while the AEs with tied weights performed best with linear activation functions. Unexpectedly, the results show that the expanders outperform the AEs.

**Discussion:**

A limitation of this research is the reliance on time series for the initial training. Future efforts will focus on developing methods that overcome this issue. Improved methods for estimating within-stride fluctuations in metabolic cost have the potential of improving rehabilitation and assistive devices by targeting the gait phases with increased metabolic cost. This research could also be applied to expand sparse measurements to locations or times that were not measured explicitly. This application would reduce the number of measurement points required to capture the response of a system.

## 1 Introduction

Impairments such as stroke, cerebral palsy, or even normal aging increase the oxygen consumption of walking (Rose et al., 1990; Platts et al., 2006). For example, on average, older adults have a 30% greater metabolic cost (Martin et al., 1992; Mian et al., 2006). When coupled with reduced oxygen uptake capacity, the ability to sustain walking becomes compromised, leading to reduced independence and quality of life (Morris and Hardman, 1997). The underlying reasons for the increased metabolic cost are inadequately understood. For example, certain training interventions (Franz, 2016) and assisting lateral balance (Ortega et al., 2008) have been ineffective in reducing metabolic costs. One potential explanation is that we cannot measure when these interventions help or hinder metabolic efficiency during the gait cycle.

Although motion capture systems can measure numerous signals with high temporal resolutions, we cannot directly measure the fluctuation of metabolic cost throughout the gait cycle. By this, we mean the fluctuation in “cost,” not “consumption.” Changes in oxygen “consumption” are slow: For example, studies show a delay with a time constant of about 40 s after an abrupt change in exercise intensity (e.g., a transition from rest to exercise) (Whipp et al., 1982; Whipp and Ward, 1990; Selinger and Donelan, 2014). Although these changes are much slower than a stride cycle (i.e., about 1 s), this does not imply that phases of the gait cycle cannot contribute by different amounts.

We define these contributions of parts of the gait cycles as “costs”. There is a consensus that different gait cycle phases have different costs (Marsh et al., 2004; Gottschall and Kram, 2005; Doke et al., 2007; Umberger, 2010; Gonabadi et al., 2020). Our current knowledge is based on indirect estimations (e.g., estimating the cost of swinging a leg while standing (Doke et al., 2007). While these estimations agree on the broad strokes (e.g., swing costs less than stance), there are large inconsistencies (Gonabadi et al., 2020; Dzewaltowski et al., 2024). This inability to estimate the cost of different phases hinders interventions: e.g., if exercise interventions or orthoses reduce metabolic cost during one phase but increase cost in another phase, we fail to understand how to improve these interventions. The present manuscript focuses on new methods for estimating the cost of different gait cycle phases.

There are several model-based methods; however, there are large differences between the used approaches, and they sometimes do not agree very well. One common approach is using musculoskeletal models and muscle metabolic rate equations. Umberger et al. (2003) developed widely used equations that estimate metabolic cost from muscle fiber work and heat energy losses associated with shortening-lengthening and activation of muscle fibers. Umberger applied his equations to a forward dynamics model with 12 muscles per side to produce the first estimation of the time series of metabolic rate during the gait cycle. Different groups developed alternative models and equations. Kim and Roberts, 2015; Roberts et al., 2016 argue that musculoskeletal models only simulate a subset of muscles, and interactions between muscles and other tissues are complex. They developed equations to estimate metabolic rate as a function of joint moments and angular velocity and produced a different time series of metabolic cost. Other estimations from Pimental et al. (2021) or Gonabadi et al. (2020) negatively correlate with the most cited time series estimation from (Umberger, 2010), which predicts that the push-off phase has the lowest cost. Some inconsistencies could be due to differences in participants, walking conditions, and measurement errors; however, it is unlikely that this explains the entire inconsistency. Reviews by Umberger and Rubenson (2011) and Hicks et al. (2015) state a need for “novel approaches.” One limitation is that no validation of the phase-specific metabolic cost has been attempted. While valuable work compared metabolic cost equations to indirect calorimetry measurements of stride mean metabolic cost (Miller, 2014; Koelewijn et al., 2019), estimations of the time series are given without validation (Umberger, 2010; Gonabadi et al., 2020; Pimentel et al., 2021).

There has been increased interest in data-driven methods that do not rely on musculoskeletal simulation. With the advent of methods and sensors that collect large human movement datasets, machine learning methods have become popular for predicting outcomes (Halilaj et al., 2018). Machine learning has already achieved very high performance in areas like interpreting histological images or estimating pressure risks (Zhang et al., 2025). Still, it is also starting to be used to estimate metabolic cost. Several regression-based and advanced machine-learning methods allowed estimating steady-state metabolic costs over shorter and shorter timescales (Selinger and Donelan, 2014). Many commercially available wearable devices (e.g., smartwatches, rings) incorporate algorithms to estimate average metabolic rate. Additionally, various research studies have developed similar algorithms for applications such as managing exercise intensity and nutritional planning. For example, a recent study uses a multi-modal algorithm based on a combination of neural networks to estimate the metabolic rate of treadmill walking in a contactless fashion using image-based sensors (Huang et al., 2024). Selinger and Donelan (2014) developed a technique that fits an exponential function to breath data to reduce the time required to predict the steady-state metabolic cost to about 2 min. Other groups developed sensor fusion methods to estimate at an even shorter timescale; Ingraham et al. (2019) evaluated different wearable sensors (respiratory, EMG, accelerometers, heart rate, and skin temperature sensors) to estimate metabolic costs. They trained different statistical models using regression and found it possible to estimate steady-state metabolic cost with a root mean square error of around 1 W kg^-1^ using 4-5 sensor modalities. In Slade et al. (2019) regression and neural network models were used to estimate steady-state metabolic cost from EMG and treadmill ground reaction forces. They could estimate the metabolic cost in a very short time (∼1 s) with an error of 8.0%.

Autoencoders (AE) are neural networks that work with machine learning and artificial intelligence. Many data science studies discuss optimization of their use, training, and performance (Vincent et al., 2010; Xie et al., 2015; Yang et al., 2016; Chai et al., 2019). In medical research, AEs are used for image processing and classification. Myronenko (2018) used an autoencoder for MRI image processing and segmentation. Ma et al. (2022) employ an enhanced edge-attention-autoencoder to improve image segmentation. Ding et al. (2019) show how image segmentation for cancer diagnosis proved reliable and faster than radiologists. AEs have also been applied in biomechanics. For example, Portnova-Fahreeva et al. (2023) employed an AE controller to perform dimensionality reduction to control a high-dimensional prosthetic hand. Huang and Zhang (2023) employed a variational AE to generate three-dimensional models of the lumbar spine for use in disease analysis and population modeling. Finally, Diethelm et al. (2024) implemented a long-short-term memory AE to detect anomalies in kinematic movement data. Dindorf et al. (2024) employed a variational AE to generate synthetic posture data for signal denoising.

The present study aims to investigate the usability of neural networks for estimating within-stride time series (such as within-stride metabolic cost) using only stride mean data as inputs. The underlying motivation is that the stride mean metabolic cost can be measured using indirect calorimetry (Margaria, 1968; Beaver et al., 1973); consequently, developing methods that estimate time series from single scalars could be useful for estimating within-stride metabolic cost. We evaluate this in a dataset with simulated walking experiments in which the metabolic cost time series can be known for training and validation. In Section 2, we discuss the datasets and models for metabolic cost and the two approaches for estimating within-stride metabolic costs. Section 3 investigates the performance of different architectures. Section 4 presents the results of the optimized networks, while Section 5 concludes with an overall discussion.

## 2 Methods

In the present study, we compare two general approaches for estimating the metabolic cost time series from the measurable mean metabolic cost: an autoencoder approach - which is implemented in two ways - and an expander approach. We trained the two autoencoders and the decoder using data from 10 model-based metabolic cost timeseries from simulated perturbed walking experiments. After training, we extracted the autoencoders and decoders to reconstruct the metabolic cost time series from an unperturbed walking condition that was left out of the training.

**FIGURE 1:**
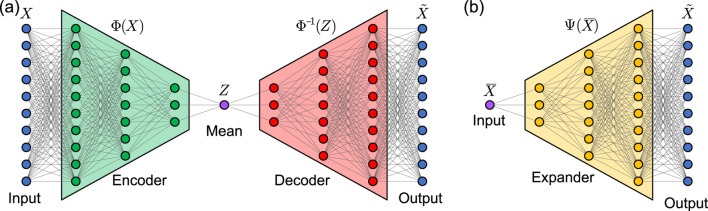
Main algorithm structures. **(a)** An autoencoder is trained to produce a time series from 1 to 100 percent of a gait cycle (e.g., the within-stride metabolic cost time series according to (Umberger, 2010)). The decoder is extracted from the autoencoder, such that it produces the estimated time series using a single value as the input (e.g., the mean metabolic cost of a gait cycle). **(b)** In a second approach, an expander network is trained directly to expand a single value to a corresponding time series (e.g., the mean metabolic cost to the corresponding within-stride metabolic cost time series).

The autoencoder approaches produce a network that maps a mean value to the corresponding time series, such that the instantaneous metabolic cost can be computed directly from the mean cost. The autoencoder (Figure 1a) takes the time series as input, encodes the mean values as the latent space, and then recovers the original time series from the latent space. After training, the decoder is extracted from the autoencoder and used separately to predict metabolic time series directly from their measured means. Our underlying reason for using this approach is the assumption that using a method that has had access to the complete timeseries as inputs could perhaps be more suitable for reconstructing timeseries than methods that never have access to timeseries as inputs. In this manuscript, we investigate this assumption by comparing the performance of autoencoder algorithms to decoder-only algorithms. We also investigated the performance of the autoencoder with both untied and tied weights between the encoder and decoder (Nowlan and Hinton, 1992). We abbreviate the untied autoencoders as UAE and the autoencoders with tied weights and bias as TAE. The expander approach leverages a single network to map a mean value to its corresponding time series, as shown in Figure 1b. In practice, the second approach is comparable to training only the decoder in the autoencoder however, we call this network an expander instead of a decoder to avoid confusion when discussing the two approaches.

Both approaches require existing time series and their mean values for training, and we rely on data from simulated walking experiments to satisfy this need. We previously generated data from simulated walking experiments (Dzewaltowski et al., 2024) using a neuromuscular simulation (Song and Geyer, 2015; Song and Geyer, 2018). The simulated walking experiments included both normal walking and 35 different cases of walking with perturbations from a robotic waist tether (Antonellis et al., 2022). The simulated data was used to compute instantaneous metabolic costs using a range of models from the literature (cf. Section 2.2), providing the necessary time series for training the two approaches. We trained the networks to reproduce the model-based within-stride metabolic costs time series using the perturbed walking conditions. We evaluated the performance of the two approaches by assessing their capability to reproduce the time series for unperturbed walking conditions (i.e., normal walking), which was not part of the training dataset.

### 2.1 Datasets

We used a neuromechanical simulation from Song and Geyer to generate data simulating walking under forced perturbations from a waist tether (Song and Geyer, 2015; Song and Geyer, 2018). Specifically, we ran a two-dimensional variant of the model that restricts motion to the sagittal plane in Simscape First Generation Multibody (MathWorks, Natick, MA). The model contained seven rigid segments simulating the legs and a trunk and 9 Hill-type muscles per leg controlled by a set of 71 muscle-reflex parameters. The 71 control parameters were optimized for each walking condition by running an optimization (Hansen, 2006) that minimized a physiologically-inspired cost function that strives to make the model walk without falling and with a minimal muscle activation sum (Dzewaltowski et al., 2024). In this framework, we simulated the effects of forward force perturbations at the hip. We simulated 32 sinusoidal force profiles with peak timings covering the entire gait cycle and peak forces ranging from 0% to 24% percent of body weight, three constant force profiles, and an unperturbed walking condition for a total of 36 conditions. After optimizing the control parameters for each walking condition, we extracted the time series to constitute the dataset for the present study. The dataset generated by this experiment is similar to the type of data that one could obtain from human motion capture experiments. For the estimation algorithm, we only used signals that are available from human motion capture experiments, such as’ strideaverage metabolic cost, joint kinematics and kinetics and muscle activations.

### 2.2 Model-based metabolic cost

We trained and evaluated our networks using 10 different model-based metabolic costs. Since the actual within-stride metabolic cost is not available, we evaluated our method “*in silico*” using simulated-within-stride metabolic cost based on methods proposed in the literature. We selected a relatively large range of model-based methods to maximize confidence in the evaluation.

We used the model-based metabolic cost methods from Bhargava, Houdijk, Lichtwark, and Umberger to generate metabolic cost time series based on force, length, and velocity time series from the muscles from the neuromuscular simulation (Bhargava et al., 2004; Lichtwark and Wilson, 2005; Houdijk et al., 2006; Umberger, 2010). Each method generates metabolic cost based on the sum of mechanical work from the muscles and energy losses from heat using slightly different proposed equations. The within-stride metabolic cost time series is produced by taking the sum of all the leg muscles. The method from Margaria estimates metabolic cost time series based on the positive and negative work (Margaria, 1968). We also used additional model-based methods from Kim and Roberts, Beck, Margaria, and Minetti to generate metabolic cost time series based on purely kinetic and kinematic data from the neuromuscular simulation (Margaria, 1968; Minetti and Alexander, 1997; Kim and Roberts, 2015; Beck et al., 2019). The method from Kim and Roberts uses joint moments and angular velocity, and we refer to the metabolic cost estimated with this model as the Kim Joint. The method from Beck uses the sum of EMG signals, which we refer to as the EMG Sum. The equation derived from Margaria was applied to joint powers and center-of-mass power similar to its implementation in (Caputo and Collins, 2014). We refer to these as Margaria Joint and Maragaria COM. The method from Minetti and Alexander estimates metabolic cost using joint moments and angular velocity, and we refer to this as Minetti Joint. Detailed explanations of the implementations are in Supplementary Material of Dzewaltowski et al. (2024).

### 2.3 Network training procedure

**FIGURE 2:**
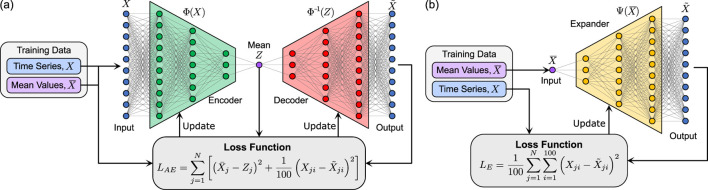
Training Process. **(a)** The training process used for the autoencoder with the loss function used. **(b)** The training process used for the expander approach with the loss function.

For both types of network approaches, the training data consists of a set of within-stride metabolic cost time series, X, and their corresponding mean metabolic cost values, $\bar{X}$ , but the two methods use this data set in different ways. For the autoencoder approach (Figure 2a), the set of within-stride metabolic cost time series, X, are passed into the encoder as input, which then compresses them down to a set of scalars for the latent space, Z . The latent space values are stored for computing a loss function and are passed to the decoder. The decoder expands the latent space into a new time series, $\tilde{X},$ that approximates the original time series. The autoencoder is trained by minimizing the following loss function

$$L_{AE}=\sum_{j=1}^{N}\left[{\left({\bar{X}}_{j}-Z_{j}\right)}^{2}+\frac{1}{100}\sum_{i=1}^{100}{\left(X_{ji}-{\tilde{X}}_{ji}\right)}^{2}\;\right],$$ (1)

where N is the number of time series in the training set (Equation 1). Note that each time series has a length of 100 units representing the gait cycle from 1% to 100% completion. The first term in the loss function computes the square error between the stride mean value and the latent space scalar, such that this causes the encoder to compress the time series to its mean value. The second term is the mean-square error computed between the original and reproduced time series, which works to make the decoder output the original time series. In the next section, we investigate the effect of network architecture, activation functions, and training epochs on the performance of the autoencoders. As mentioned previously, we evaluate the performance of the autoencoder without and with tied weights between the encoder and decoder. In both cases, autoencoders are trained using the Adam optimization algorithm, which is a stochastic gradient descent method that uses adaptive estimates of the first- and second-order moments (Kingma and Ba, 2014). Probably should say what the default values are, and what package/library/code language was used to make the AI models.

For the expander (Figure 2b), the set of mean values, $\bar{X}$ , are passed into the expander as input and expanded into a new time series, $\tilde{X}$ . The loss function is

$$L_{E}=\frac{1}{100}\sum_{j=1}^{N}\sum_{i=1}^{100}{\left(X_{ji}-{\tilde{X}}_{ji}\right)}^{2},$$ (2)

which is the mean-square error between the original and reproduced time series where N is the number of time series in the training set (Equation 2). The minimization of the loss, $L_{E}$ , causes the expander to output time series that are representative of those provided in the training set. The expander is also trained using the Adam optimization algorithm (Kingma and Ba, 2014).

### 2.4 Network application procedure for estimating the within-stride time series

**FIGURE 3:**
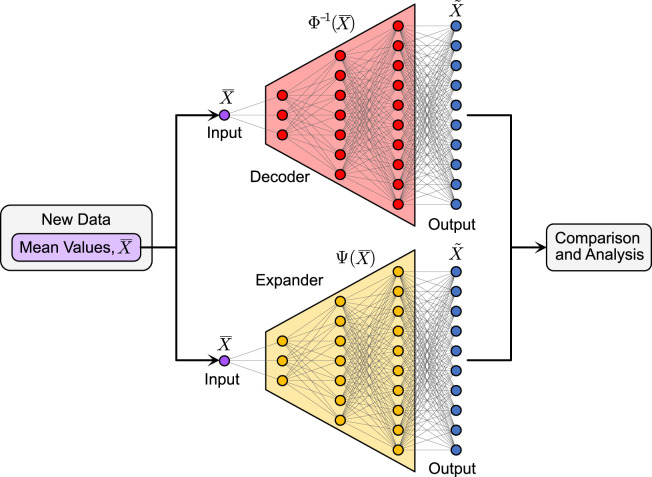
Application Process. In the application step, the stride mean value of an unknown time series (e.g., within-stride metabolic cost) is passed as input to the decoder or expander to estimate the corresponding time series. Note that the decoders and expanders are applied separately using the same input data.

The three networks (two autoencoders and one expander) were trained using the 10 metabolic cost models computed from the simulated walking data for only the perturbed walking conditions. The metabolic costs for the unperturbed walking condition were reserved for validation of the trained networks. After training, the decoders were extracted from the autoencoders to identify the metabolic time series from a given stride mean value. The expander was used in the same way as the extracted decoders. Figure 3 presents the application process of the two methods, which shows how the mean value is used as the only input to either network to identify the corresponding time series. After the training is completed, both approaches can estimate within-stride metabolic cost time series without needing time series as inputs.

### 2.5 Tuning of network architecture

We evaluated different architectures and compared their performance by computing the Pearson correlation coefficient, r, between the actual within-stride metabolic cost and the one estimated by the network. For our data, p<0.05 when r>0.1966 , such that a high correlation coefficient corresponds to a significant finding. As a result, the correlation coefficient is sufficient for making decisions on the performance of each model. We first consider the effect of the number of layers on the performance of each network for up to three hidden layers, as listed in Table 1. We use an arithmetic sequence to reduce the number of neurons from 100 (the length of the metabolic cost time series) to 1 neuron (the length of the mean metabolic cost) for the encoders, and the decoders and expanders use the reverse sequence. Note that the encoders also have an input layer with 100 neurons (not included in Table 1). We set all activation functions to linear for this study, the number of epochs to 2000, and repeat the training process 50 times for each architecture. We only consider three layers at most because the results for higher layer counts will be the same as three layers due to the use of linear activation functions. The number of epochs was chosen based on preliminary experiments with the networks, and a study on the effect of the number of epochs was performed after determining the final network architecture. Repeating the training process 50 times using new networks each time allows us to investigate the repeatability of the results. At the start of each iteration, the networks from the previous iteration are deleted, and new networks are created to ensure that they start with new initial weights and biases. At the end of each iteration, we save the networks and their training history, compute and store r for each metabolic cost, and then delete the networks. After training each network, we compute the average r value for each of the 10 metabolic costs across the 50 cases and use that as the metric for comparison. The networks are trained using the dataset of the perturbed walking conditions and then applied to the data from the unperturbed walking condition for evaluation.

**TABLE 1:** Network Architectures. The architectures listed in the table are used to study the effect of the number of layers on the performance of each network.

Hidden Layers	UAE and TAE neurons		Expander Neurons
	Encoder	Decoder	
1	100, 1	1, 100	1,100
2	100, 50, 1	1, 50, 100	1, 50, 100
3	100, 66, 33, 1	1, 33, 66, 100	1, 33, 66, 100

**FIGURE 4:**
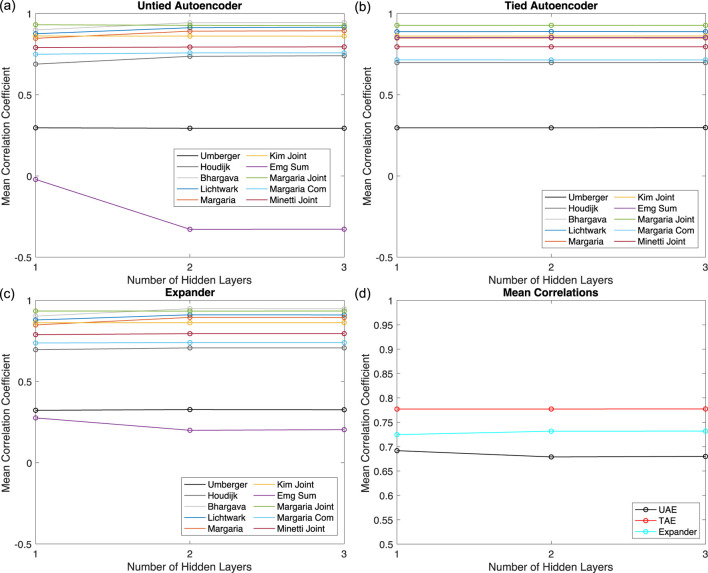
Network Architecture Results. The mean r values for each metabolic cost for **(a)** the UAEs, **(b)** the TAEs, and **(c)** the expanders. **(d)** The mean r value across all metabolic costs for the three networks.

The results of the network architecture study are presented in Figures 4a–c for the UAE, TAE, and expander, respectively, for the ten metabolic costs. The results show that the UAE and expander networks converge to the same result when two or more hidden layers are included as expected. The TAE networks appear to converge to the same values regardless of the number of layers, but there are small variations in the r values that are not visible on the scale used in the figures. However, those variations are small enough to conclude that the TAE produces nearly the same results regardless of the number of layers. This suggests that tying the parameters leads to a more efficient deconstruction and reconstruction of the time series. The UAE and expander networks perform the worst for the EMG Sum metabolic cost, and all three networks perform relatively poorly for the Umberger metabolic cost. Figure 4d presents the mean r across the ten metabolic costs for each network, which shows that the TAE performs better than the expander and UAE on average under these conditions. Furthermore, this figure demonstrates that all three networks converge at two or fewer layers.

We proceeded with studying the effect of the activation functions on each network’s performance to improve the results for the EMG Sum and Umberger metabolic costs while maintaining the results for the others. We consider six nonlinear activation functions: relu, elu, sigmoid, silu, mish, and tanh. We start with networks with two layers and change the activation function in the layer with 50 neurons. The reason we modify this layer is that the networks need to end with a linear activation function to ensure that the output is scaled appropriately for each metabolic cost. Additionally, some of the metabolic cost models (e.g., Margaria) had negative values for some portions of the gait cycle, which cannot be captured using nonlinear activation functions selected as they converge to fixed values for negative inputs. Just as in the study of the number of layers, we set the number of epochs to 2000, repeat the training process 50 times for each activation function, and then compute the mean r value for each metabolic cost across the 50 trials.

**FIGURE 5:**
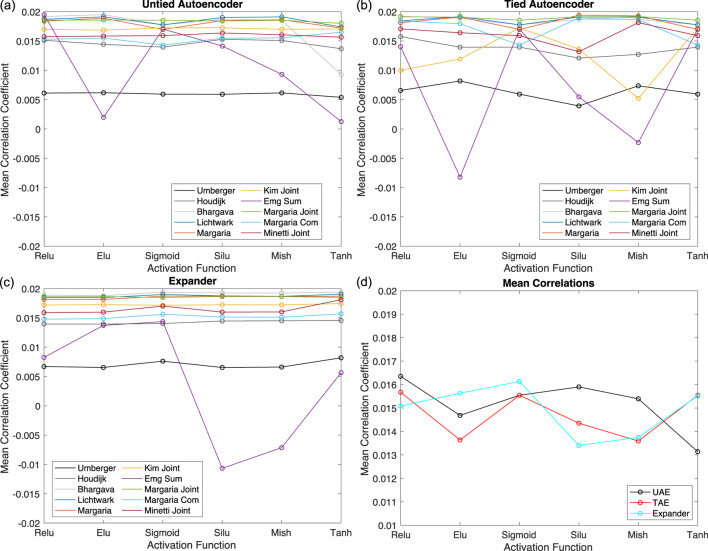
Activation Function Results for Two Hidden Layers. The mean r values for each metabolic cost for different activation functions and two hidden layers for **(a)** the UAEs, **(b)** the TAEs, and **(c)** the expanders. **(d)** The mean r value across all metabolic costs for the three networks for different activation functions and two hidden layers.

We depict the results for two layers in Figures 5a–c for the UAEs, TAEs, and expanders. We also present the average r value across the ten metabolic costs for each network in Figure 5d. Regardless of the nonlinear activation function chosen, all three networks perform significantly worse than when only linear activation functions were used.

**FIGURE 6:**
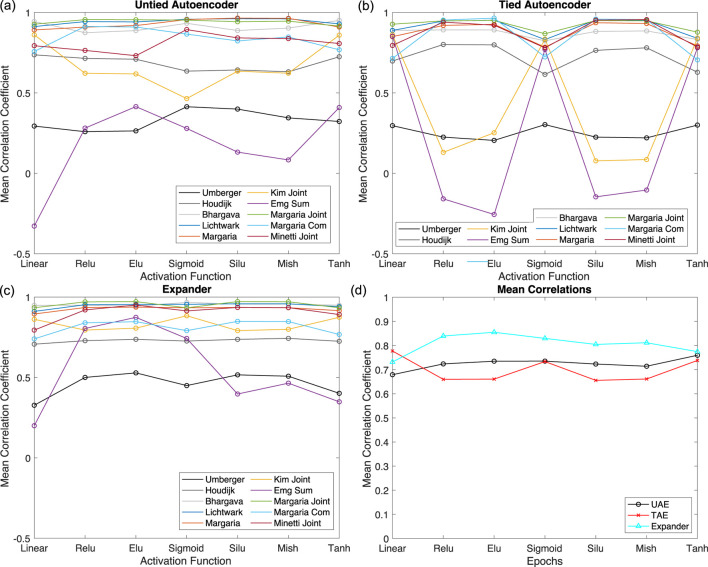
Activation Function Results for Three Hidden Layers. The mean r values for each metabolic cost for different activation functions and three hidden layers for **(a)** the UAEs, **(b)** the TAEs, and **(c)** the expanders. **(d)** The mean r value across all metabolic costs for the three networks for different activation functions and three hidden layers.

Based on these results, we switch to the three-layer networks and modify the layer’s activation function with 66 neurons, such that the nonlinear activation function is sandwiched between two layers with linear activation functions. We perform the same study as with the two-layer network with 2000 epochs and 50 trials and present the results in Figures 6a–c for the UAEs, TAEs, and expanders. We also include the performance of the case where a linear activation function is used in the layer with 66 neurons. Figure 6d presents the mean r values across all ten metabolic costs for each network and provides an estimation of the overall performance of each network for each nonlinear activation function.

For the UAEs, the nonlinear activation functions have the biggest effect on model fit for EMG Sum and Kim Joint metabolic costs but have a relatively small effect on the others, including the Umberger metabolic cost that we want to improve. The tanh activation function results in the best overall performance, as seen in Figure 6d. For the TAEs, the nonlinear activation functions have the largest influence on the EMG Sum and Kim Joint metabolic costs, just like the UAEs. An interesting pattern appears where nonlinear activation functions that worsen the EMG Sum, Kim Joint, and the Umberger metabolic costs improve the results for the other metabolic costs, and *vice versa*. However, the improvement gained for the EMG Sum, Kim Joint, and Umberger metabolic costs is substantially larger than the decrease seen for the other metabolic costs. From Figure 6d, the TAEs perform the best when only linear activation functions are used. For the expanders, we find that all nonlinear activation functions improve the performance of the networks overall compared to the linear activation functions. The EMG Sum and Umberger metabolic costs peak for the elu activation function, and only a small decrease in performance for the Kim Joint is observed for this function. Indeed, Figure 6d shows that using the elu activation function results in the best performance for the expanders.

**FIGURE 7:**
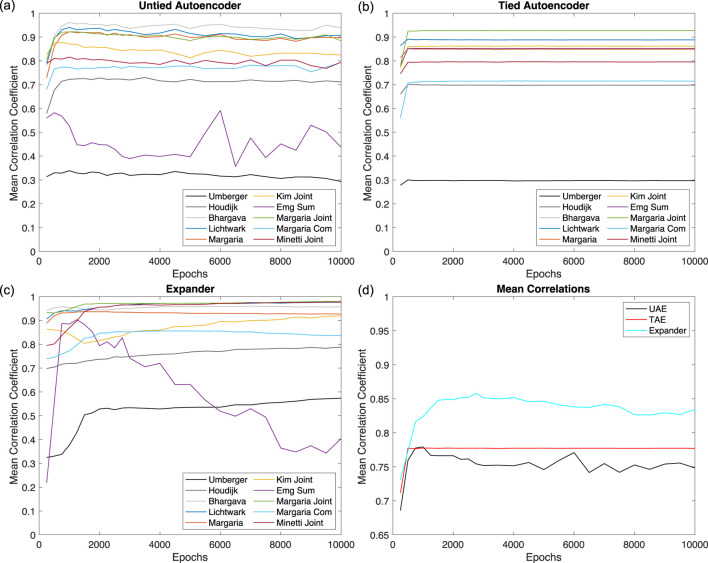
Effect of Training Epochs. Correlation coefficients as a function of epochs. Results indicate that the optimal number of epochs is approximately 2000. **(a)** Untied autoencoder. **(b)** Tied Autoencoder. **(c)** Expander. **(d)** Mean correlations.

Based on these results, we fix the number of layers to three and set the activation function in the layer with 66 neurons to tanh, linear, and elu for the UAEs, TAEs, and expanders, respectively. We then proceed with investigating the effect of the number of epochs on the performance of each network using 50 trials just as in the previous studies. We consider the performance of the networks for epochs ranging from 250 to 3,000 with a step size of 250 and also from 3,500 to 10,000 with a step size of 500. We present the results for the UAEs, TAEs, and expanders in Figures 7a–c, respectively, and the mean r values across all metabolic costs are presented in Figure 7d. For the UAEs, we find that the performance for most of the metabolic cost models improves before plateauing as the number of epochs increases, except for the EMG Sum model. The UAEs perform poorly for the Umberger model regardless of the number of epochs. For the EMG Sum model, the UAEs perform well up to 1,000 epochs before dropping in performance and spiking up again at 6,000 epochs. The best performance for the UAEs occurs for 1,000 epochs as seen in Figure 7d. The TAEs depict relative independence with respect to the number of epochs due to the use of only linear activation functions and converge to their optimal results after 500 epochs. The performance of the expanders increases with the number of epochs except for the EMG Sum model, where the performance initially increases before decreasing. The expanders exhibit optimal performance when the number of epochs is set to 2,750, but at this value the performance for the EMG Sum is relatively weak compared to lower values. For example, using 2000 epochs, the overall results are comparable with those for 2,750, but the EMG Sum performance is significantly better. Interestingly, expanders perform better than the other two for fewer epochs. The UAE and TAE perform similarly for their optimal number of epochs, which suggests that there is an upper limit on the performance of an autoencoder for this problem. Based on these results, we conclude that the optimal networks are three layers where the middle layer uses tanh, linear, and elu for the activation functions with the numbers of epochs set to 1,000, 500, and 2000 for the UAE, TAE, and expanders, respectively.

## 3 Results

**FIGURE 8:**
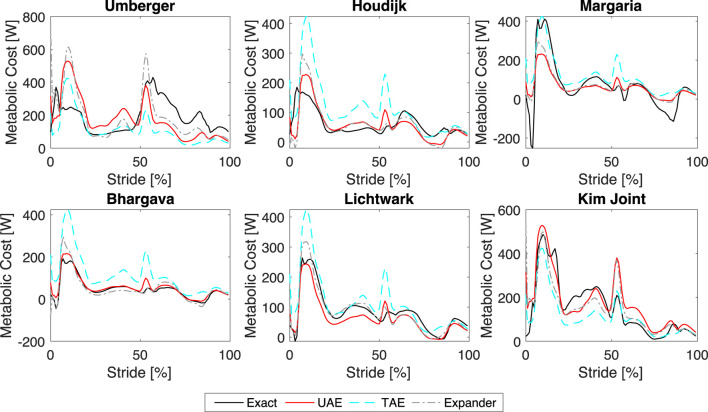
Estimated versus Actual Metabolic Cost Time Series. Comparison of the exact and estimated metabolic cost time series for the Umberger, Houdijk, Margaria, Bhargava, Lichtwark, and Kim Joint models.

**FIGURE 9:**
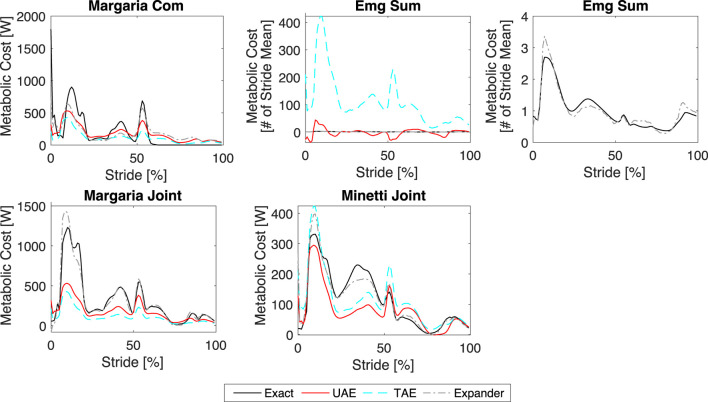
Results. Comparison of the exact and estimated metabolic cost time series for the Margaria COM, EMG Sum, Margaria Joint, and Minetti Joint models.

Using the best-performing network configurations as discussed in the previous section, we trained each network using the perturbed datasets and then estimated the metabolic cost time series for the unperturbed dataset using the corresponding mean metabolic costs as input. We present the exact and estimated metabolic cost time series for Umberger, Houdijk, Margaria, Bhargava, Lichtwark, and Kim Joint models in Figure 8. We present the remaining models (Margaria COM, EMG Sum, Margaria Joint, and Minetti Joint) in Figure 9. The EMG Sum is provided twice: once with the predictions from all networks and a second time with only the estimation from the expander, which shows that only the expander can reproduce the EMG Sum model. We provide the r values for each network and metabolic cost model in Table 2. We find that none of the networks can accurately reproduce the Umberger metabolic cost, whereas all three do a good job reproducing the Bhargava, Lichtwark, Kim Joint, and Margaria Joint models. The expander performs better than the other two networks for the Umberger, Houdijk, Lichtwark, Margaria, EMG Sum, Margaria Joint, Margaria COM, and the Minetti Joint models. Importantly, the expander is the only network that reproduces both the shape and the amplitude of the EMG Sum model, which confirms that the process used to determine the number of layers, activation functions, and number of epochs was successful.

**TABLE 2:** Correlation coefficients. The r values for the results provided in Figures 8, 9 (MC is metabolic cost models).

MC	UAE	TAE	EX	MC	UAE	TAE	EX
Umberger	0.339	0.297	0.544	Kim Joint	0.874	0.863	0.783
Houdijk	0.722	0.697	0.741	EMG Sum	0.493	0.852	0.963
Bhargava	0.963	0.889	0.940	Margaria Joint	0.927	0.928	0.972
Licthwark	0.947	0.888	0.954	Margaria COM	0.769	0.717	0.843
Margaria	0.919	0.849	0.937	Minetti Joint	0.804	0.797	0.939

**FIGURE 10:**
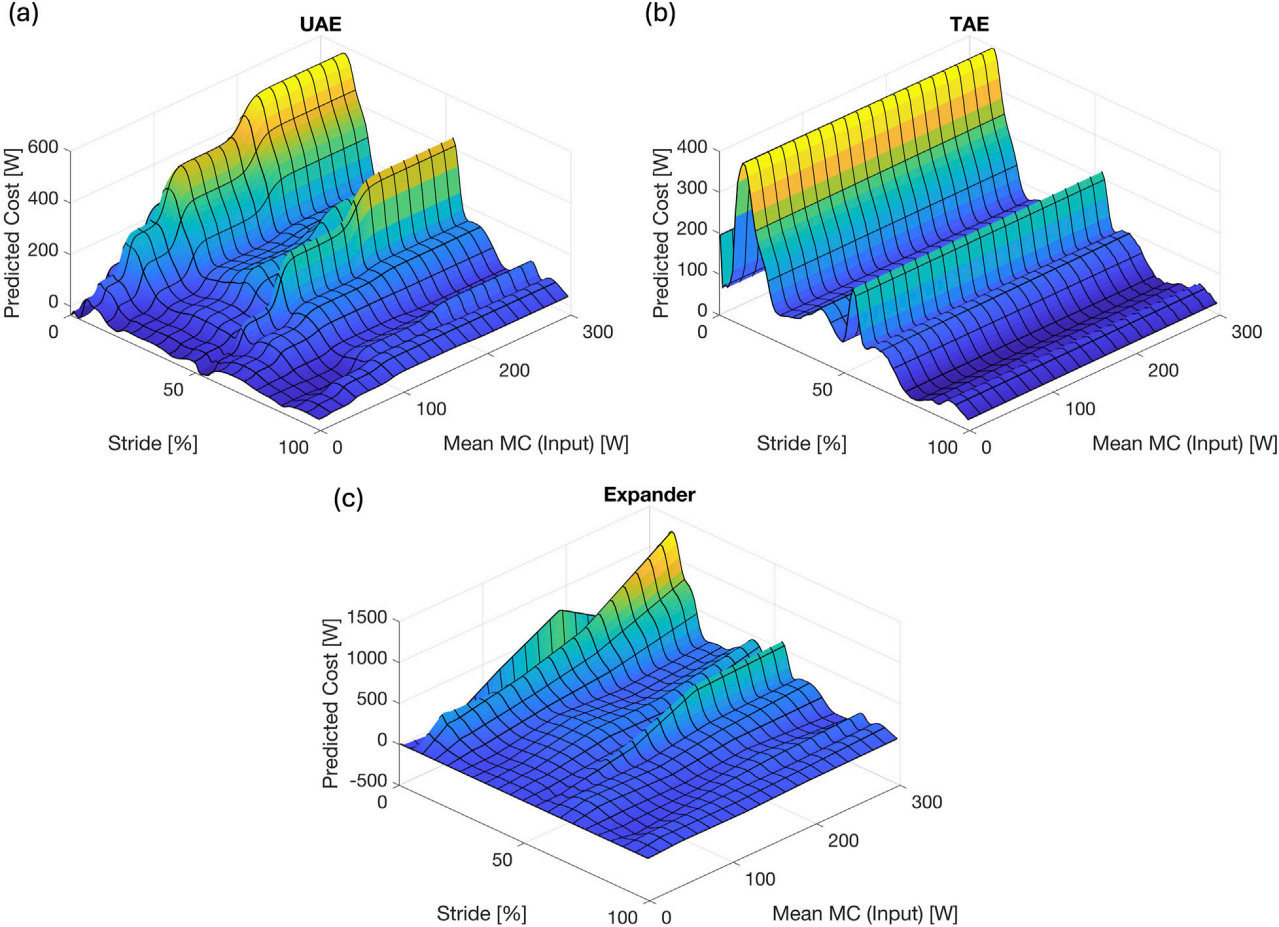
Surface Profiles. Surface profiles for **(a)** the UAE network, **(b)** the TAE network, and **(c)** the expander network.

Next, we consider the mapping from mean metabolic cost to instantaneous cost produced by each network by plotting their outputs as surfaces in Figures 10a–c for the UAE, TAE, and expander, respectively. The surface outputs are computed for mean metabolic costs varying from 0 to 300 W. The surface profile produced by the UAE reveals that the output clusters to three regimes that are connected by step-ups in amplitude. Interestingly, the increases in amplitude occur for all portions of the stride at the same mean metabolic costs, though the amounts of increase vary. To investigate these results, we trained five different UAE networks and compared their surface profiles (not shown here). We found that the stepped-surface profile is a generic result for the UAEs, though the locations and widths of the steps varied for each network. Furthermore, we found that all parts of the profile increased at the same mean metabolic cost values just as seen in Figure 10a, such that this is also a generic result for the UAE.

Interestingly, time series estimated by the TAE appear to have the same shape with only minor differences in amplitude. Several possible explanations exist for this result: first, the TAE may not have enough trainable parameters to adequately capture the variations across the different metabolic cost models; second, the use of linear activation functions causes the TAE to only be able to identify a best approximation of all models; or third, the mapping of instantaneous cost to mean cost is non-invertible and a different model is required to map the mean cost to the instantaneous cost, which cannot be achieved with the TAE due to the tied weights and biases. For the first possible explanation, the TAE has 19310 trainable parameters for this configuration while the UAE and expanders have 28021 and 9010, respectively. Thus, the TAE clearly has enough trainable parameters to capture the variations across the different models. To explore the second reason, we replaced the nonlinear activation functions in the UAE and expander networks with a linear activation function, and then computed their corresponding surface profiles (not shown here). We found that both the UAE and expander networks increased monotonically in instantaneous amplitude as the mean metabolic cost increased, such that they were able to capture the variations in metabolic cost unlike the TAE. Thus, the use of linear activation functions is not the reason for the lack of variation in the TAE surface profile. Instead, we conclude that the mapping from instantaneous to mean metabolic cost is non-invertible and a different mapping is needed to Convert from mean cost to instantaneous cost. This is further supported by the fact that the UAE can reproduce the metabolic cost models better than the TAE across the range of mean costs.

Overall, the surface profile for the expander shows the greatest variation including both increases and decreases in amplitudes as the mean metabolic cost increases. Furthermore, the changes in amplitude do not occur uniformly across the gait cycle unlike the profile for the UAE. Thus, the use of the elu nonlinear activation functions gives the expanders the ability to adapt their amplitudes at different stages of the gait cycle independently for a given mean metabolic cost input. These results suggest that learning the full time series from its mean value is an easier problem to solve than mapping the time series to a mean value then expanding that learned mean back to the time series. This is interesting because one would expect that incorporating the original time series into the network as in the UAE and TAE would produce better results than providing only the mean as the input as in the expander. Furthermore, these results support the conclusion that training a network to map instantaneous metabolic cost to mean produces a model that is non-invertible, and a different model is needed to map the mean cost back to the time series.

## 4 Discussion

This research investigated the performance of two approaches for estimating the within-stride metabolic cost of the gait cycle directly from the measurable mean metabolic cost. The first approach employed autoencoders to train a decoder to map the mean value to the corresponding time series. The second approach trained an expander network to directly produce the instantaneous metabolic cost from the corresponding mean value. The networks were applied to walking data generated using a neuromechanical simulation under 35 different forced perturbations applied through a waist tether and one unperturbed state. The networks were trained using the perturbed walking datasets then applied to the unperturbed data and the results were evaluated using the Pearson correlation coefficient.

The networks were constructed using an arithmetic sequence from 100 to 1 neuron for the encoders and expanders and the reverse sequence was used for the decoders and expanders. The effect of the number of layers (and number of neurons due to the arithmetic sequence), activation functions, and the number of epochs on the performance of each network were investigated using trials of 50 different networks in each study. The investigation concluded that 3 layers with a nonlinear activation function sandwiched between two linear layers produced the best results for the UAE and expanders, while using only linear activation functions for the TAE networks performed best. The best nonlinear activation function for the UAE was determined to be the tanh function while the elu function was best for the expanders. The optimal number of epochs was found to be 1,000, 500, and 2000 for the UAEs, TAEs, and expanders, respectively.

The results revealed that the UAE and expander were able to reproduce a wider range of metabolic cost models whereas the TAE converged to a single profile that best approximates all models losing the ability to capture the variations of individual models. Looking into the individual series models results, the UAE and TAE performed poorly for the Umberger and EMG Sum models failing to reconstruct the data with acceptable accuracy, whereas the expander showed a more precise reproduction for the same models and a noticeable enhancement for the EMG Sum estimation. Of note, the Umberger model is one of the five models that calculate metabolic cost based on kinetic and kinematic data from the internal muscles of the model (Dzewaltowski et al., 2024). While internal muscle data was used for evaluating the metabolic cost estimation, it was not included as an input to the estimation (training) algorithms to realistically reflect that internal muscle data would not be available during human experiments. The fact that the algorithms did not have access to the source data of the Umberger metabolic cost model could explain the inadequate performance in reconstructing the Umberger model series.

As for the rest of the metabolic cost models, namely, Kim Joint, EMG Sum, Margaria Joint, Margaria COM, and Minetti Joint, these metabolic cost models were based on motion capture and EMG signals. The simulated data for such signals was used as inputs to the estimation algorithms since it is typically available and measurable in human experiments. This probably explains the relatively better evaluation results, except for EMG Sum. We believe that the relatively worse evaluation result for estimating the EMG Sum based metabolic cost could probably be explained by the relatively noisier nature of this metric.

Overall, the expanders performed consistently better than the autoencoders, such that the expanders are recommended for use over the autoencoders. The reasons behind that are related to the fact that the autoencoders compression stage results in significant losses of details in data series leading to a propagation of these losses in the reconstruction stage. To achieve this compression, generally a significant number of hidden layers have to be added into the construction of the auto-encoder with careful selection of activation functions to ensure the accuracy of the compression-reconstruction (Osaulenko, 2021). Expanders on the other hand work better when it comes to series reconstruction especially for complex data series with subtle variations (Prabhu et al., 2017), as seen in the metabolic cost times series. The major strength of the approaches considered here, especially with the expander, is that the networks can produce the instantaneous metabolic cost directly from the mean metabolic cost, providing a window into how different parts of the gait cycle can vary in cost. Once trained, the application of the networks is fast, such that they could be used for quick diagnostics or real time applications, such as energy expenditure estimation during exercise.

One of the limitations of the employed approaches is that they rely on simulated time series for training and optimizing the network parameters. As such, the output of the networks cannot be regarded as the true metabolic cost, but rather a representation of the real cost based on a nonlinear combination of the models used in the training. A related limitation is that this entire research study is done on a simulated walking dataset. Even though this entire research study is done on a simulated walking dataset(s), this method offers an advantage for *in silico* validation research since the ground truth metabolic cost is known because it is defined during the generation of the simulation. We acknowledge the importance of experimental validation, and to that end our group is actively working on collecting data for human perturbation experiments as well as the recently published walking dataset (Antonellis et al., 2022; Dzewaltowski et al., 2024). Additionally, creative validation approaches for testing whether estimation methods can reproduce model-based metabolic costs (Dzewaltowski et al., 2024) or evaluating whether the estimation methods can detect induced changes like increased metabolic cost of the swing phase.

Another limitation with the architecture tuning is that only linear layers were used. While the tuning did show some architectures performed better than others the finding of no benefit of adding more than two hidden layers could potentially be explained by this. The performance of the networks could be improved by enriching the dataset with additional models for metabolic cost as well as new datasets generated from enhanced computational models. However, this limitation does not prevent one from applying the networks to study how different physical changes (e.g., aging or therapeutic devices) alter the instantaneous metabolic costs as well as the costs incurred during specific phases of the gait cycle. Improving the confidence and knowledge of the within-stride fluctuations in metabolic cost could be useful for rehabilitation and assistive devices for clinical populations. More specifically this knowledge could enable design rehabilitation interventions that specifically target the costliest phase of the gait cycle and assistive devices that focus assistance during the costliest phase of the gait cycle. Another limitation is that the high performance of the trained networks could be due to an already strong correlation and similarity between the perturbed and unperturbed walking datasets. As such, the results and performance of the networks could be improved by providing a wider range of perturbed conditions using different physical alterations (e.g., perturbing the ankle instead of the waist). Future efforts are focused on creating methods that estimate within-stride metabolic costs that do not rely on those datasets in the training process as well as on the application of the networks discussed here for determining how different physical conditions alter specific phases of the gait cycle. Additionally, further work will focus on leveraging multiple methods for estimating instantaneous metabolic cost to cross-validate the methods while also identifying changes to the cost in specific phases of the gait cycle.

## Data Availability

The original contributions presented in the study are included in the article/Supplementary Material, further inquiries can be directed to the corresponding author.
