# Supplementary figures and images for: Estimating within-stride metabolic cost from stride-average data using autoencoders and expander networks
Source: Front Bioeng Biotechnol. 2025 Jun 20;13:1579085. doi: 10.3389/fbioe.2025.1579085 (PMC12226529; doi:10.3389/fbioe.2025.1579085)

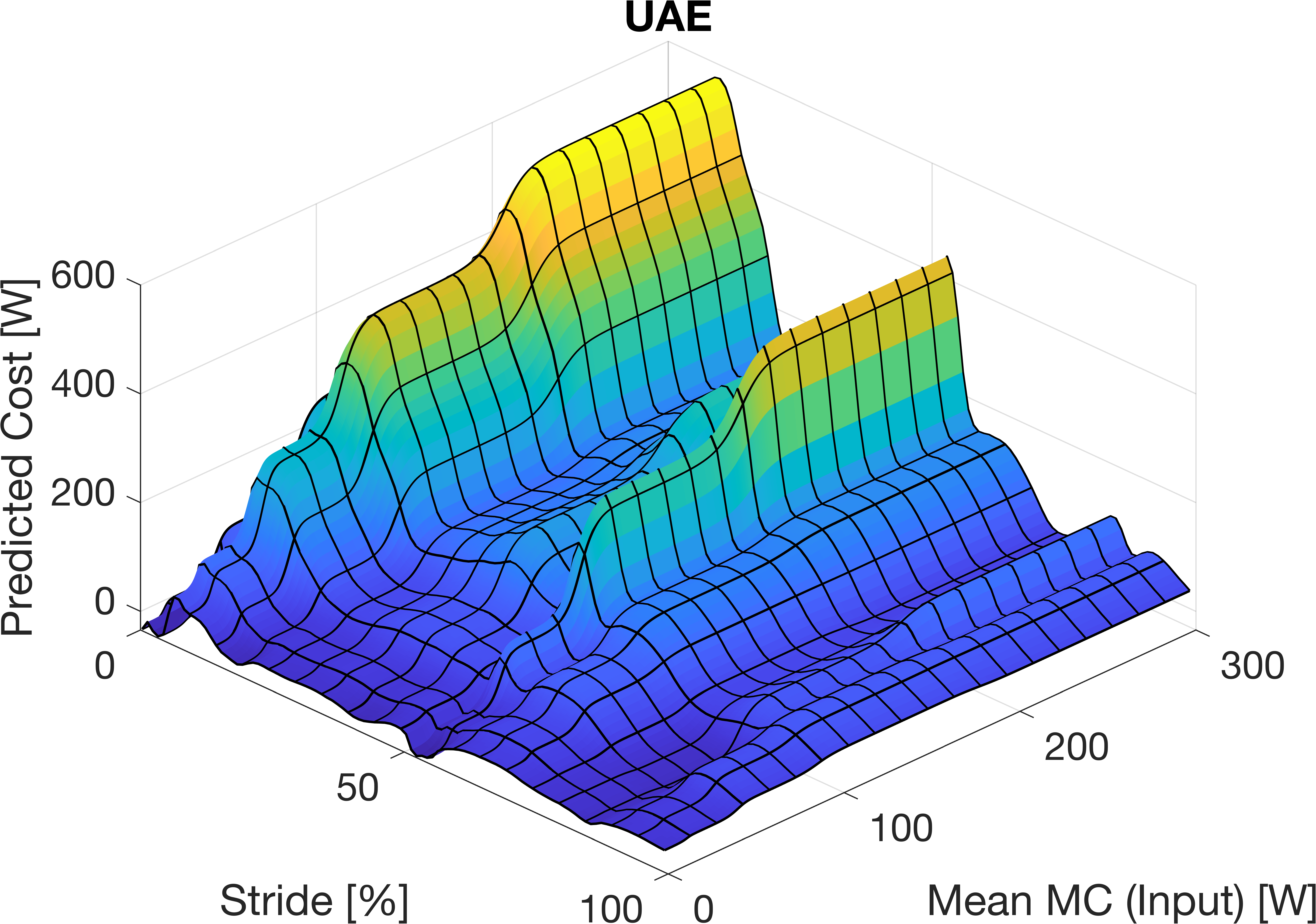

Supplement: Supplementary file 1 [file Datasheet1.ZIP › SuppMat/UAESurface.png]

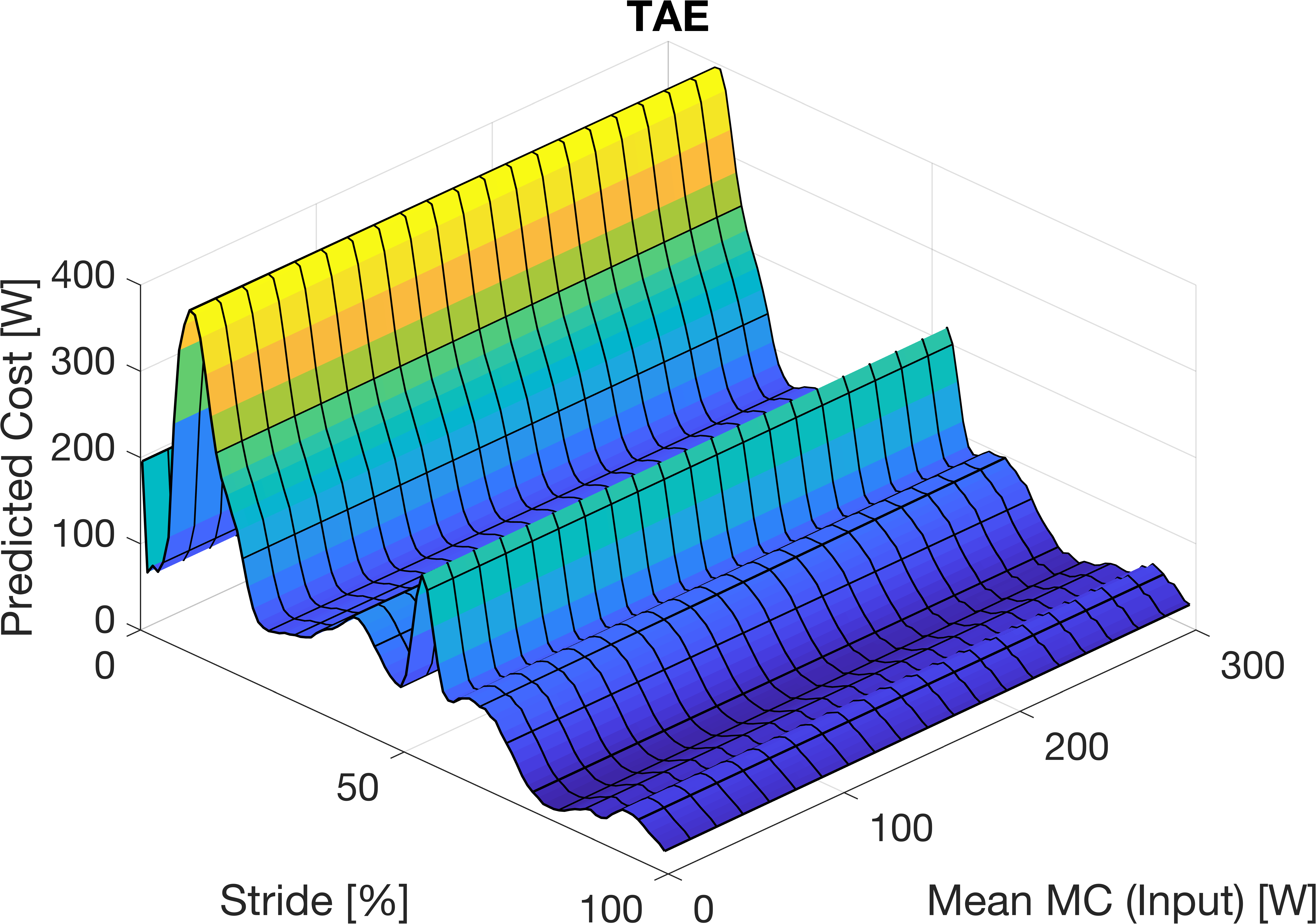

Supplement: Supplementary file 1 [file Datasheet1.ZIP › SuppMat/TAESurface.png]

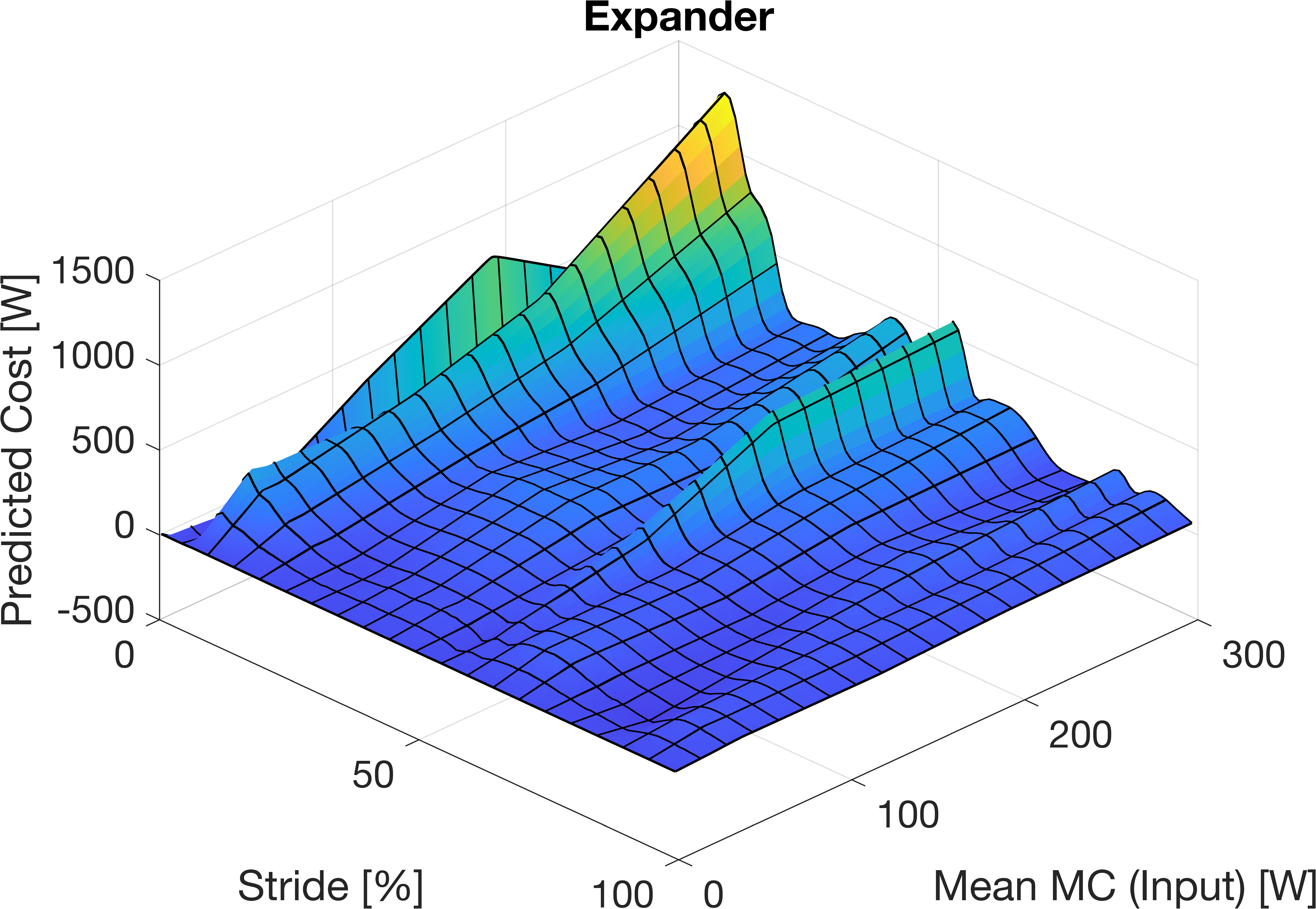

Supplement: Supplementary file 1 [file Datasheet1.ZIP › SuppMat/ExSurface.png]
